# Evidence for the Role of Mast Cells in Cystitis-Associated Lower Urinary Tract Dysfunction: A Multidisciplinary Approach to the Study of Chronic Pelvic Pain Research Network Animal Model Study

**DOI:** 10.1371/journal.pone.0168772

**Published:** 2016-12-21

**Authors:** Xu Wang, Wujiang Liu, Michael O'Donnell, Susan Lutgendorf, Catherine Bradley, Andrew Schrepf, Liwei Liu, Karl Kreder, Yi Luo

**Affiliations:** 1 Department of Urology, University of Iowa, Iowa City, Iowa, United States of America; 2 Tianjin Institute of Urology, The 2^nd^ Hospital of Tianjin Medical University, Tianjin Medical University, Tianjin, China; 3 Department of Psychology, University of Iowa, Iowa City, Iowa, United States of America; 4 Department of Obstetrics and Gynecology, University of Iowa, Iowa City, Iowa, United States of America; Cedars-Sinai Medical Center, UNITED STATES

## Abstract

Bladder inflammation frequently causes cystitis pain and lower urinary tract dysfunction (LUTD) such as urinary frequency and urgency. Although mast cells have been identified to play a critical role in bladder inflammation and pain, the role of mast cells in cystitis-associated LUTD has not been demonstrated. Interstitial cystitis/bladder pain syndrome (IC/BPS) is a chronic and debilitating inflammatory condition of the urinary bladder characterized by the hallmark symptoms of pelvic pain and LUTD. In this study we investigated the role of mast cells in LUTD using a transgenic autoimmune cystitis model (URO-OVA) that reproduces many clinical correlates of IC/BPS. URO-OVA mice express the membrane form of the model antigen ovalbumin (OVA) as a self-antigen on the urothelium and develop bladder inflammation upon introduction of OVA-specific T cells. To investigate the role of mast cells, we crossed URO-OVA mice with mast cell-deficient *Kit*^*W-sh*^ mice to generate URO-OVA/*Kit*^*W-sh*^ mice that retained urothelial OVA expression but lacked endogenous mast cells. We compared URO-OVA mice with URO-OVA/*Kit*^*W-sh*^ mice with and without mast cell reconstitution in response to cystitis induction. URO-OVA mice developed profound bladder inflammation with increased mast cell counts and LUTD, including increased total number of voids, decreased mean volume voided per micturition, and decreased maximum volume voided per micturition, after cystitis induction. In contrast, similarly cystitis-induced URO-OVA/*Kit*^*W-sh*^ mice developed reduced bladder inflammation with no mast cells and LUTD detected. However, after mast cell reconstitution URO-OVA/*Kit*^*W-sh*^ mice restored the ability to develop bladder inflammation and LUTD following cystitis induction. We further treated URO-OVA mice with cromolyn, a mast cell membrane stabilizer, and found that cromolyn treatment reversed bladder inflammation and LUTD in the animal model. Our results provide direct evidence for the role of mast cells in cystitis-associated LUTD, supporting the use of mast cell inhibitors for treatment of certain forms of IC/BPS.

## Introduction

Interstitial cystitis/bladder pain syndrome (IC/BPS) is a chronic inflammatory condition of the urinary bladder characterized by the hallmark symptoms of pelvic pain and lower urinary tract dysfunction (LUTD) such as urinary frequency and urgency [1]. IC/BPS patients exhibit an increased number of mast cells in the bladder and elevated levels of mast cell mediators in the urine such as interleukin (IL)-6, nerve growth factor (NGF), histamine/methyhistamine, and tryptase [2,3]. Since these mediators are vasoactive, nociceptive and pro-inflammatory, mast cells are considered to play an important role in the pathophysiology of IC/BPS [2]. Many factors including neuropeptides (e.g. substance P and neurotensin), growth factors (e.g. NGF), and cytokines (e.g. tumor necrosis factor-α and stem cell factor) can activate mast cells to release mediators [2]. Clinical trials in IC/BPS using mast cell inhibitors have been conducted and demonstrated to be promising [4–6]. In line with human studies, animal studies have demonstrated that mast cells are responsible for bladder inflammation and pain in diverse models [7–10]. However, despite these studies, the role of mast cells in cystitis-associated LUTD has not been identified.

The etiology of IC/BPS remains elusive and may involve multiple causes. Although autoimmunity is debated as a potential cause of IC/BPS, evidence suggests that it may play an important role in the pathophysiology of the disease. It has been reported that IC/BPS patients develop antinuclear and anti-urothelium autoantibodies [11], overexpress urothelial HLA-DR molecules [12], and coexist with other autoimmune diseases such as Sjogren’s syndrome, systemic lupus erythematosus, and rheumatoid arthritis [1,11,13,14]. Moreover, immunosuppressive drugs have been used to treat IC/BPS and demonstrated to be beneficial for patients [15,16]. Furthermore, bladder histopathology data have revealed a role of cell-mediated immune mechanisms in IC/BPS [17,18]. Hence, autoimmune inflammation is likely a component of the pathophysiology in subgroups of IC/BPS patients.

Animal models with bladder autoimmune inflammation have been actively used in IC/BPS research for over two decades [10,19–24]. Experimental autoimmune cystitis (EAC) can be induced through immunization with bladder tissue components in rodents. These EAC models have been used to reproduce many clinical correlates of IC/BPS, offering a unique property for investigation of specific aspects of the disease such as cystitis pain and voiding dysfunction [10,23,24]. Using genetic engineering technology, we previously developed a transgenic EAC model (URO-OVA) that expresses the membrane form of the model antigen ovalbumin (OVA) as a self-antigen on the urothelium and develops bladder inflammation at 7–14 days after adoptive transfer of OVA-specific T cells for cystitis induction [25,26]. The URO-OVA model resembles many clinical features of IC/BPS including increased bladder mast cell counts, pelvic pain, and LUTD [25,27]. Here we used this transgenic EAC model to identify the role of mast cells in cystitis-associated LUTD. Our results provide direct evidence for the role of mast cells in LUTD in the animal model, supporting the use of mast cell inhibitors for treatment of certain forms of IC/BPS.

## Materials and Methods

### Ethics statement

All animal experiments were approved by University of Iowa Animal Care and Use Committee (Permit Number: 1308153) and performed according to the Guide for the Care and Use of Laboratory Animals of the National Institutes of Health. Animals were euthanized via CO_2_ asphyxiation, followed by physical confirmation of euthanasia.

### Mice

URO-OVA mice were previously developed in our laboratory [25]. Mast cell-deficient B6.Cg-*Kit*^*W-sh*^ mice (*Kit*^*W-sh*^) were obtained from Jackson Laboratories (Bar Harbor, ME). URO-OVA/*Kit*^*W-sh*^ mice were generated through crossbreeding of the two strains and selected as described previously [25,28]. URO-OVA/*Kit*^*W-sh*^ mice (homozygous) retained urothelial OVA expression but lacked endogenous mast cells. OT-I mice that express CD8^+^ T cell receptor (Vα2Vβ5) specific for the H2-K^b^/OVA_257–264_ epitope were used to provide OVA-specific CD8^+^ T cells for cystitis induction [25,29]. All mice were housed in a pathogen-free facility at the University of Iowa Animal Care Facility.

### Mast cell reconstitution

Bone marrow cells were prepared from the femurs of C57BL/6 mice (8 weeks old) and cultured in RPMI 1640 medium supplemented with 10% fetal bovine serum, penicillin (100 units/ml), streptomycin (100 μg/ml), β-mercaptoethanol (50 μM), and mouse recombinant IL-3 (20 ng/ml; eBioscience, San Diego, CA) for 5 weeks [28]. The purity of mast cell cultures was >95% as verified by toluidine blue staining (S1 Fig). URO-OVA/*Kit*^*W-sh*^ mice (6 weeks old) were intravenously (i.v.) injected with 1 X 10^6^ mast cells and used 9 weeks after mast cell reconstitution for cystitis induction.

### Cystitis induction

Age- and sex-matched URO-OVA and URO-OVA/*Kit*^*W-sh*^ mice were injected i.v. with OT-I splenocytes (5 X 10^6^ cells per mouse) that had been pre-activated with OVA_257–264_ peptide in culture for 3 days as described previously [25]. Mice were then evaluated for voiding habits and bladder inflammation at days 6 and 7 after cystitis induction, respectively.

### Mast cell blockage

URO-OVA mice (8 weeks old) were injected intraperitoneally (i.p.) with cromolyn sodium (Sigma, St Louis, MO), a mast cell membrane stabilizer [30], at 10 mg/kg/dose daily beginning one day before cystitis induction up to day 13 after cystitis induction (S2 Fig). Control mice were treated i.p. with saline. Mice were induced for cystitis as described above and evaluated for voiding habits at day 13 (after last cromolyn dose) and bladder inflammation at day 14 after cystitis induction.

### Bladder histology

Bladders were collected and processed for formalin fixation, paraffin embedment, section preparation, hematoxylin and eosin (H&E) staining, and photography as described previously [25]. Bladder inflammation was scored in a blinded manner based on infiltration of inflammatory cells in the lamina propria and the presence of interstitial edema as described previously: 1+ (mild infiltration with no or mild edema), 2+ (moderate infiltration with moderate edema), and 3+ (moderate to severe infiltration with severe edema) [25]. Mast cells were stained using toluidine blue solution [31] and counted in a blinded manner.

### Voiding habit analysis

As described previously [32], mice were placed in individual micturition cages (Columbus Instruments, Columbus, OH) for 24-hour real time recording of voiding habits with 12-hour light and 12-hour dark cycles. Mice had free access to drinking water but were restrained from solid food to prevent feces from interfering with the measurement of urine output. The system was computer interfaced for automated data acquisition in 2-minute intervals using Oxymax software (Columbus Instruments, Columbus, OH). Urinary frequency, voided volume per micturition, and total urine volume were recorded.

### Flow cytometry

Bladder single-cell suspensions were prepared, stained with FITC-CD8 and/or PE-Vα2 antibodies (eBioscience, San Diego, CA), and analyzed by flow cytometry as described previously [25].

### Reverse Transcriptase-Polymerase Chain Reaction (RT-PCR)

Bladder total RNAs were extracted using Qiagen RNeasy Mini Kit (Qiagen, Valencia, CA) and cDNAs were synthesized using Invitrogen SuperScript III Reverse Transcriptase (Invitrogen, Carlsbad, CA) and Oligo dT as described previously [25]. PCR amplification was performed on cDNA products using Taq DNA polymerase (New England Biolabs, Ipswich, MA) and sequence-specific primer pairs for interferon (IFN)-γ (5’-TGAACGCTACACACTGCATCT-3’ and 5’-GACTCCTTTTCCGCTTCCTGA-3’; 459 bp), IL-6 (5’-GTTCTCTGGGAAATCGTGGA-3’ and 5’-GGAAATTGGGGTAGGAAGGA-3’; 339 bp), tumor necrosis factor (TNF)-α (5’-CGTCAGCCGATTTGCTATCT-3’ and 5’-CGGACTCCGCAAAGTCTAAG-3’; 206 bp), NGF (5’-CTGTGGACCCCAGACTGTTT-3’ and 5’-CACTGAGAACTCCCCCATGT-3’; 194 bp), tachykinin-1 (SP precursor) (5’-GCCAATGCAGAACTACGAAA-3’ and 5’-GCTTGGACAGCTCCTTCATC-3’; 280 bp), and glyceraldehyde-3-phosphate dehydrogenase (GAPDH) (5’-GTTCCAGTATGACTCCACT-3’ and 5’-GTGCAGGATGCATTGCTG-3’; 321 bp). Based on our pre-established PCR kinetics, GAPDH was amplified for 25 cycles, IFN-γ, TNF-α, NGF and SP precursor were amplified for 36 cycles, and IL-6 was amplified for 40 cycles. The PCR products were run on 1% agarose gels, stained with ethidium bromide, and imaged by Gel Doc EZ Imager (Bio-Rad Laboratories, Hercules, CA).

### Statistical analysis

Results were analyzed using GraphPad Prism (Version 6.0g) and presented as mean ± s.d. for mast cells and CD8^+^Vα2^+^ cells in the bladder and voiding habit changes after cystitis induction. Data was compared using Student’s *t*-test (two groups) or ANOVA followed by LSD post hoc tests (multiple groups). A value of *p*<0.05 was considered statistically significant.

## Results

### Mast cell-deficient URO-OVA/*Kit*^*W-sh*^ mice develop reduced bladder inflammation after cystitis induction

**Fig 1:**
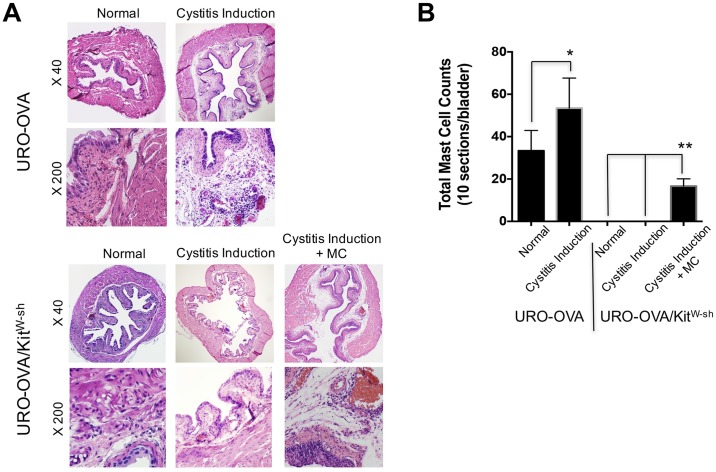
Mast cell-deficient URO-OVA/*Kit*^*W-sh*^ mice developed reduced bladder inflammation after cystitis induction. (A) At day 7 after cystitis induction the bladders of URO-OVA and URO-OVA/*Kit*^*W-sh*^ mice (both with and without mast cell reconstitution) were collected, sectioned, and analyzed by histological H&E staining. The normal bladders of URO-OVA and URO-OVA/*Kit*^*W-sh*^ mice were included for comparison. Magnifications: X40 and X200. The images are representative of 6 bladders per group. (B) The bladder sections were stained with toluidine blue solution and mast cells were counted in 10 consecutive sections for each bladder. *p<0.05 and **p<0.01. MC, mast cells.

URO-OVA mice developed bladder inflammation at day 7 after cystitis induction as described previously [25,33,34]. The inflamed bladders exhibited prominent cellular infiltration, edema, and hyperemia (Fig 1A, top panel; Table 1, score: 3+) as well as increased mast cell counts (Fig 1B; S3 Fig). In contrast, mast cell-deficient URO-OVA/*Kit*^*W-sh*^ mice developed reduced bladder inflammation (Fig 1A, bottom panel; Table 1, score: 0–1+) with no mast cells detected (Fig 1B; S3 Fig). However, URO-OVA/*Kit*^*W-sh*^ mice restored the ability to develop bladder inflammation after reconstitution with mast cells (Fig 1A, bottom panel; Table 1, score: 2–3+). Scattered mast cells were detected in the inflamed bladders of mast cell-reconstituted URO-OVA/*Kit*^*W-sh*^ mice (Fig 1B; S3 Fig). These observations indicate that mast cells are indispensable for the development of bladder inflammation in the URO-OVA model.

**Table 1:** Bladder response to cystitis induction.

	Bladder Inflammatory Score			
	-	+	++	+++
**URO-OVA**				
Normal (n = 6)	6			
Cystitis induction (n = 6)				6
**URO-OVA/*Kit***^***W-sh***^				
Normal (n = 6)	6			
Cystitis induction (n = 6)	1	5		
Cystitis induction + MC (n = 6)			2	4

MC = mast cells

### Mast cell-deficient URO-OVA/*Kit*^*W-sh*^ mice exhibit no significant changes in voiding habits after cystitis induction

**Fig 2:**
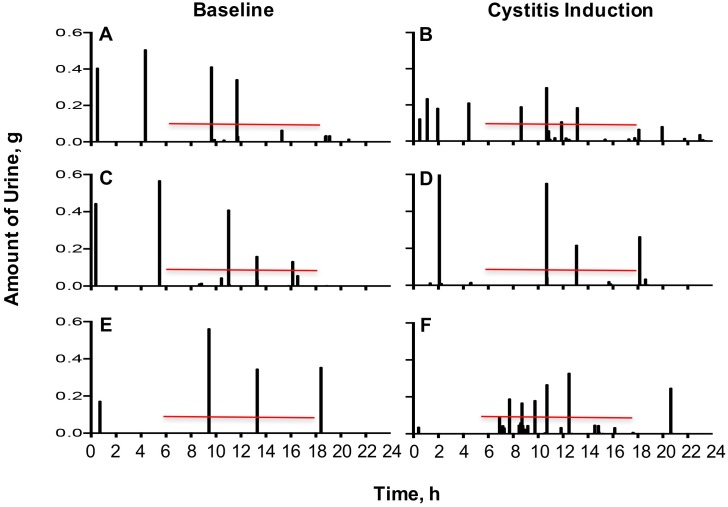
Mast cell-deficient URO-OVA/*Kit*^*W-sh*^ mice exhibited no significant changes in voiding habits after cystitis induction. At day 6 after cystitis induction URO-OVA mice (B), URO-OVA/*Kit*^*W-sh*^ mice (D), and mast cell-reconstituted URO-OVA/*Kit*^*W-sh*^ mice (F) were placed in micturition cages for 24-hour micturition recording (see Table 2). The baseline voiding habits of URO-OVA (A), URO-OVA/*Kit*^*W-sh*^ (C), and mast cell-reconstituted URO-OVA/*Kit*^*W-sh*^ mice (E) were included for comparison. Data are shown as the amount (gram) of urine collected in 2-minite intervals during the 24-hour period. The results are representative of 5 mice for each of the three groups. The dark period is indicated by red lines.

To identify whether mast cells play a role in cystitis-associated LUTD, we placed URO-OVA/*Kit*^*W-sh*^ mice (both mast cells reconstituted and non-reconstituted), together with URO-OVA mice, in micturition cages for a 24-hour recording of voiding habits at day 6 after cystitis induction. Baseline voiding habits were recorded for comparison. As observed previously [27], URO-OVA mice developed significant changes in voiding habits after cystitis induction (Table 2). These changes included decreased mean volume voided per micturition (*p* = 0.0053), decreased maximum volume voided per micturition (*p* = 0.012), and increased total number of voids (*p* = 0.0002). The number of voids in the dark period was increased (*p* = 0.0107). Unlike URO-OVA mice, URO-OVA/*Kit*^*W-sh*^ mice developed no significant changes in voiding habits after cystitis induction (Table 2). However, after reconstitution with mast cells, URO-OVA/*Kit*^*W-sh*^ mice restored the ability to develop LUTD (*p* = 0.0391 for decreased mean volume voided per micturition, *p* = 0.0301 for decreased maximum volume voided per micturition, and *p* = 0.0354 for increased total number of voids) (Table 2). Excessive urination in the dark period was also observed in mast cell-reconstituted URO-OVA/*Kit*^*W-sh*^ mice, although it did not reach statistical significance (*p* = 0.0638) (Table 2). Fig 2 represents the typical voiding habits of URO-OVA (Fig 2A and 2B), URO-OVA/*Kit*^*W-sh*^ (Fig 2C and 2D), and mast cell-reconstituted URO-OVA/*Kit*^*W-sh*^ mice (Fig 2E and 2F) before (baseline) and after cystitis induction. The total voided volumes in 24 hours were similar between before and after cystitis induction (URO-OVA mice: 1.721±0.4851 vs. 1.349±0.4104 g, *p* = 0.2271; URO-OVA/*Kit*^*W-sh*^ mice: 1.483±0.5591 vs. 1.766±0.5236 g, *p* = 0.4333; mast cell-reconstituted URO-OVA/*Kit*^*W-sh*^ mice: 1.737±0.4901 vs. 1.383±0.4353 g, *p* = 0.2607). These observations indicate a critical role of mast cells in cystitis-associated LUTD in the URO-OVA model.

**Table 2:** Changes in voiding habits after cystitis induction.

	Baseline	Cystitis Induction	*p-value
**URO-OVA (n = 5)**			
Mean volume voided per micturition (g)	0.275 ± 0.09	0.113 ± 0.03	0.0053
Maximum volume voided per micturition (g)	0.566 ± 0.099	0.26 ± 0.098	0.012
Total number of voids (in dark)	6.4 ± 0.89 (2.6 ± 1.1)	11.6 ± 1.5 (6.4 ± 2.3)	0.0002 (0.0107)
**URO-OVA/*Kit***^***W-sh***^ **(n = 5)**			
Mean volume voided per micturition (g)	0.275 ± 0.112	0.228 ± 0.06	0.4333
Maximum volume voided per micturition (g)	0.621 ± 0.065	0.611 ± 0.177	0.9033
Total number of voids (in dark)	6.2 ± 2.95 (4.6 ± 2.2)	8.0 ± 2.24 (4.6 ± 1.5)	0.3085 (>0.9999)
**URO-OVA/*Kit***^***W-sh***^ **+ MC (n = 5)**			
Mean volume voided per micturition (g)	0.27 ± 0.087	0.136 ± 0.033	0.0391
Maximum volume voided per micturition (g)	0.574 ± 0.079	0.365 ± 0.135	0.0301
Total number of voids (in dark)	6.6 ± 1.6 (4.2 ± 1.3)	12.3 ± 3.1 (9.3 ± 4.5)	0.0354 (0.0638)

MC = mast cells;

*p = compared to Baseline

### Blocking mast cell activation reverses bladder inflammation and voiding dysfunction in URO-OVA mice after cystitis induction

**Fig 3:**
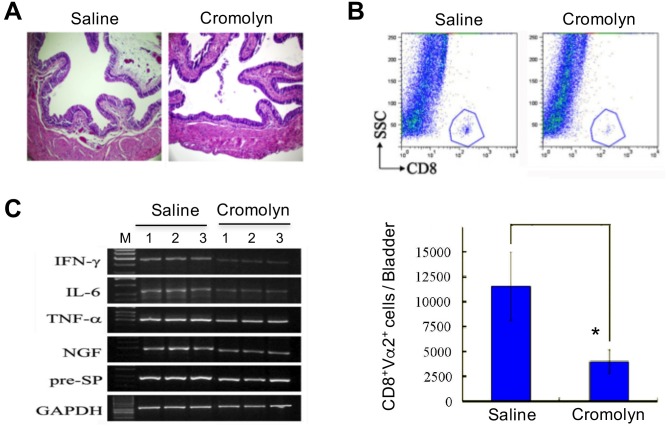
Cromolyn treatment reverses bladder inflammation in URO-OVA mice after cystitis induction. Mice were treated with saline or cromolyn daily beginning one day before cystitis induction up to day 13. The bladders were collected at day 14 and processed for histological H&E staining (A), flow cytometric analysis of bladder infiltrating CD8^+^ T cells and CD8^+^Vα2^+^ T cells (B), and RT-PCR analysis of mRNAs for inflammatory factors IFN-γ, IL-6, TNF-α, NGF and substance P precursor (pre-SP) (C). GAPDH was used as an internal control. M, a 100 bp DNA marker. *p<0.05. The images are representative of 8 bladders per group.

To conform the role of mast cells in bladder inflammation and associated LUTD, we treated URO-OVA mice with cromolyn or control saline during the development of bladder inflammation in the animal model (S2 Fig). Compared to saline-treated mice, cromolyn-treated mice exhibited no or marginal bladder inflammation (Fig 3A) with substantially reduced infiltrating CD8^+^ and CD8^+^Vα2^+^ T cells at day 14 after cystitis induction (Fig 3B). The bladders of cromolyn-treated mice also expressed reduced levels of mRNAs for mast cell- and sensory neuron-derived inflammatory factors IFN-γ, IL-6, TNF-α, NGF and substance P precursor (Fig 3C). In parallel with reduced bladder inflammation, the cromolyn-treated mice exhibited no significant changes in voiding habits compared to baseline voiding habits at day 13 after cystitis induction (Table 3). In contrast, saline-treated mice exhibited no improvement in voiding dysfunction compared to baseline voiding habits at the same conditions (Table 3; *p*<0.0001 for both mean and maximum volumes voided per micturition and *p* = 0.0035 for total number of voids). There were also significant differences between cromolyn- and saline-treated groups in voiding habits (Table 3; *p* = 0.0057 for mean volume voided per micturition, *p*<0.0001 for maximum volume voided per micturition, and *p* = 0.0198 for total number of voids). The total voided volumes in 24 hours were similar between normal, cystitis-induced plus saline-treated, and cystitis-induced plus cromolyn-treated groups (1.561±0.3821 vs. 1.039±0.1675 vs. 1.398±0.4281 g, *p* = 0.2739). These observations indicate that blocking mast cell activation reverses bladder inflammation and voiding dysfunction in the URO-OVA model.

**Table 3:** Cromolyn treatment reverses voiding dysfunction in URO-OVA mice after cystitis induction.

	Baseline	Cystitis Induction + Saline	Cystitis Induction + Cromolyn
**Mean volume voided per micturition (g)**	0.272 ± 0.06	0.106 ± 0.036 *p<0.0001	0.258 ± 0.127 *p = 0.7795 **p = 0.0057
**Maximum volume voided per micturition (g)**	0.590 ± 0.093	0.191 ± 0.033 *p<0.0001	0.599 ± 0.115 *p = 0.875 **p <0.0001
**Total number of voids**	6.0 ± 1.852	10.50 ± 3.117 *p = 0.0035	6.375 ± 3.159 *p = 0.7763 **p = 0.0198

n = 8 mice/group;

*p = compared to Baseline;

**p = compared to Cystitis Induction + Saline group

## Discussion

Mast cells are considered to play an important role in IC/BPS, as many patients exhibit increased mast cell counts in the bladder and elevated levels of mast cell mediators in the urine [2,3]. To support the human study observations, animal studies have demonstrated that mast cells are indispensable for the development of bladder inflammation and pain seen in IC/BPS patients [7–10]. However, despite these observations, the role of mast cells in cystitis-associated LUTD has not been identified. In this study we used a transgenic EAC model (URO-OVA), which resembles many clinical features of IC/BPS including increased bladder mast cell counts, pelvic pain, and voiding dysfunction [25,27], to investigate whether mast cells play a role in cystitis-associated LUTD.

To facilitate investigation of the role of mast cells, we crossed URO-OVA mice with mast cell-deficient *Kit*^*W-sh*^ mice to generate mast cell-deficient URO-OVA/*Kit*^*W-sh*^ mice. We observed that URO-OVA/*Kit*^*W-sh*^ mice developed reduced bladder inflammation with no detectable LUTD after cystitis induction. However, reconstitution of mast cells in URO-OVA/*Kit*^*W-sh*^ mice restored their ability to develop bladder inflammation and LUTD in response to cystitis induction. In addition, treatment of URO-OVA mice with systemic cromolyn, a mast cell membrane stabilizer [30], reversed bladder inflammation and LUTD in this model. Our observations indicate that mast cells play a critical role in cystitis-associated LUTD in the URO-OVA model.

Although multiple treatment modalities are currently available for IC/BPS patients, they are largely empirical and often dissatisfactory and vary in efficacy. Therefore, effort to develop new therapies for IC/BPS is greatly needed. Since mast cells are considered to play a critical role in the pathophysiology of IC/BPS, pharmacotherapy with drugs specific for mast cell inhibition may benefit IC/BPS patients. In addition to traditionally used antihistamine drugs [35], inhibitors directly targeting mast cells have been tested and demonstrated to be promising in IC/BPS clinical trials [4–6]. In this study we have observed that treatment with systemic cromolyn effectively inhibit bladder inflammation and LUTD in the IC/BPS-like URO-OVA model. Hopefully, therapies directed toward inhibiting mast cells may yet prove effective in the treatment of IC/BPS patients.

We previously demonstrated that the URO-OVA model was responsive to intravesical treatment with anti-inflammatory agents such as dimethyl sulfoxide [33] and RDP58 [34]. We observed that both agents inhibited bladder inflammation through suppressing T cell activity and/or viability in the animal model. Since these anti-inflammatory agents and mast cell inhibitory agents (e.g. cromolyn) act through different pathways, a combination therapy with the two agent types may result in improved effects on treating bladder inflammation and LUTD in IC/BPS patients. We plan to test this combination therapy using the URO-OVA model in the near future.

The present study demonstrates the role of mast cells in LUTD at an acute cystitis setting. We will extend to identify the role of mast cells at a chronic cystitis setting to more closely mimic human IC/BPS. Our future studies will also include to identify which mast cell mediator(s) play a predominant role in cystitis-associated LUTD in the URO-OVA model and use this model to develop new therapies for LUTD and pain seen in IC/BPS patients.

## Conclusions

Our study provides direct evidence for the role of mast cells in cystitis-associated LUTD, supporting the use of mast cell inhibitors for treatment of certain forms of IC/BPS.

## Supporting Information

S1 Fig Toluidine blue staining of bone marrow-derived mast cells in culture. Magnifications: X200 and X1000.
(TIF)

S2 Fig Experimental schedule for cystitis induction, cromolyn treatment, and biological analysis in URO-OVA mice. (TIF)

S3 Fig Toluidine blue staining of mast cells in the bladders. Mast cells were detected in both URO-OVA and mast cell-reconstituted URO-OVA/*Kit*^*W-sh*^ mice but not in URO-OVA/*Kit*^*W-sh*^ mice at day 7 after cystitis induction. Mast cells are indicated by red arrows. MC, mast cells. Magnification: X400.
(TIF)
