# Supplementary figures and images for: Evidence for the Role of Mast Cells in Cystitis-Associated Lower Urinary Tract Dysfunction: A Multidisciplinary Approach to the Study of Chronic Pelvic Pain Research Network Animal Model Study
Source: PLoS One. 2016 Dec 21;11(12):e0168772. doi: 10.1371/journal.pone.0168772 (PMC5176179; doi:10.1371/journal.pone.0168772)

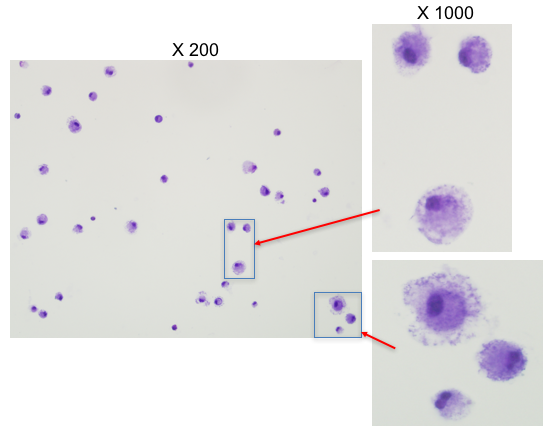

Supplement: S1 Fig — Magnifications: X200 and X1000. (TIF) [file pone.0168772.s001.tif]

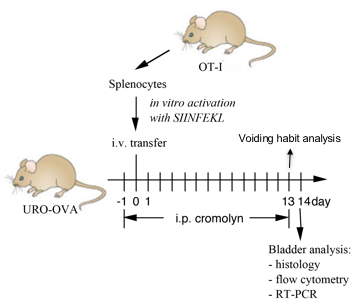

Supplement: S2 Fig — (TIF) [file pone.0168772.s002.tif]

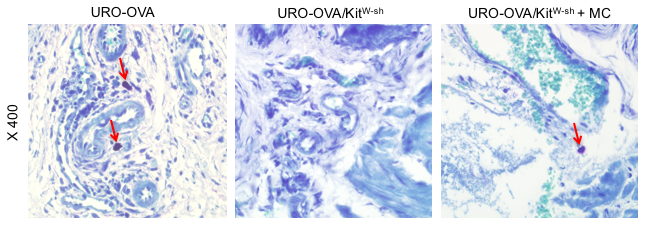

Supplement: S3 Fig — Mast cells were detected in both URO-OVA and mast cell-reconstituted URO-OVA/KitW-sh mice but not in URO-OVA/KitW-sh mice at day 7 after cystitis induction. Mast cells are indicated by red arrows. MC, mast cells. Magnification: X400. (TIF) [file pone.0168772.s003.tif]
